# Supplementary material for: Genome-Wide Identification and Expression Analysis of UBiA Family Genes Associated with Abiotic Stress in Sunflowers (Helianthus annuus L.)
Source: Int J Mol Sci. 2023 Jan 18;24(3):1883. doi: 10.3390/ijms24031883 (PMC9916351; doi:10.3390/ijms24031883)
Supplement: Supplementary file 1 [file ijms-24-01883-s001.zip › ijms-2126980-supplementary.pdf]

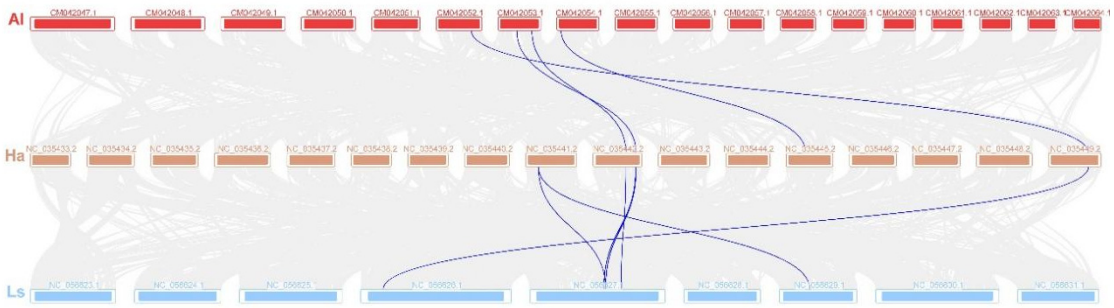

Figure S1. Collinearity analysis of *UBiA* gene in Asteraceae

UBiA genes of *Helianthus annuus*, *Arctium lappa*, and *Lactuca sativa* are covariate. The red color is the *Arctium lappa* chromosome, the brown color is the *Helianthus annuus* chromosome, and the blue color is the *Lactuca sativa* chromosome.

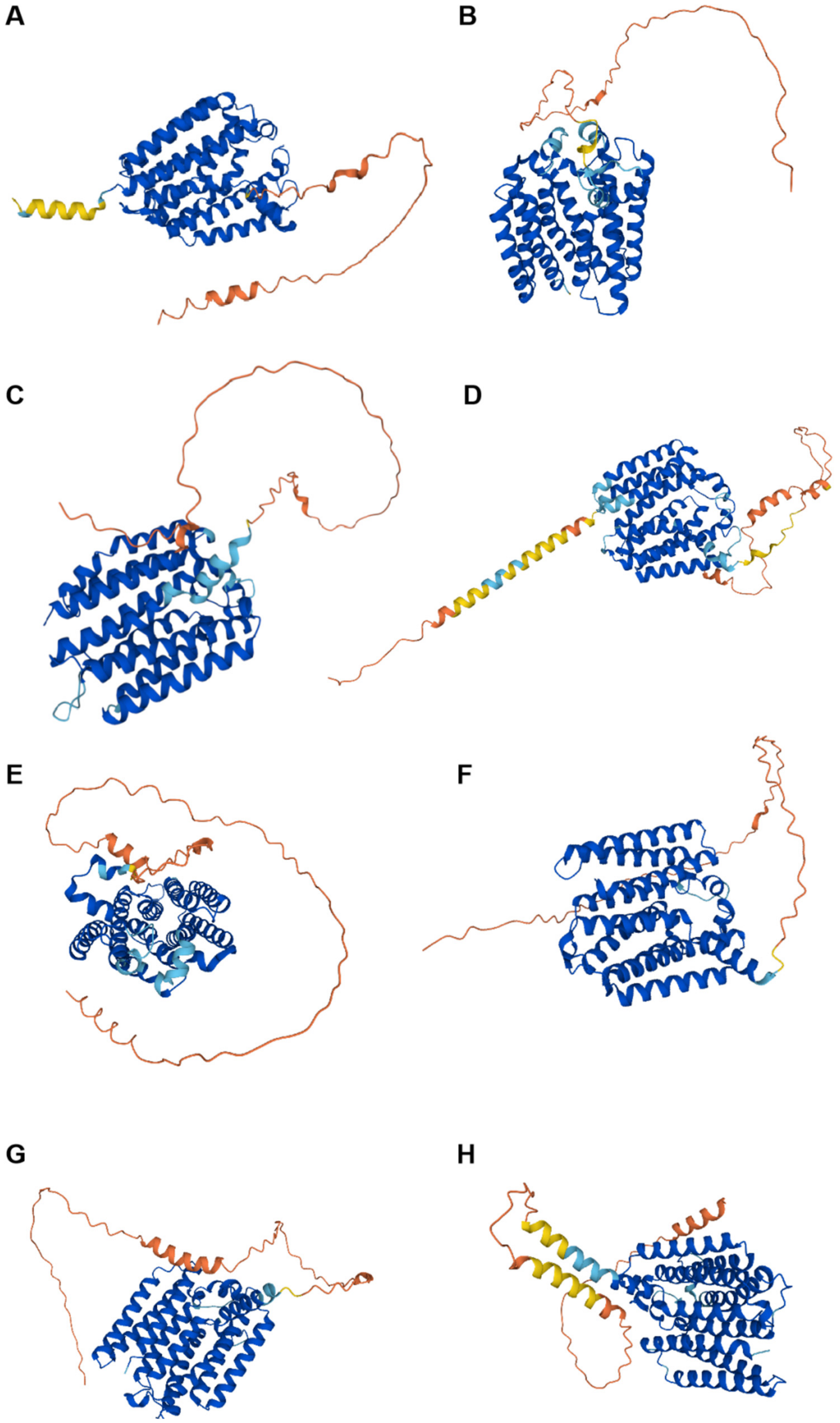

Figure S2. Schematic diagram of UBiA structural domain

A. PHB polyprenyltransferase (PPT), B. Chlorophyll synthases (ATG4), C. DHNA-phytyltrans Ferases (ABC4), D. Protoheme IX farnesyltransferases (COX10), E. Homogentisate phytyltransferase (HPT), F. Homogentisate solanesyl transferase (HST), G. 4-dimethylallytransferase (G4DT), H. Homogentisate geranylgeranyl transferase (HGGT).

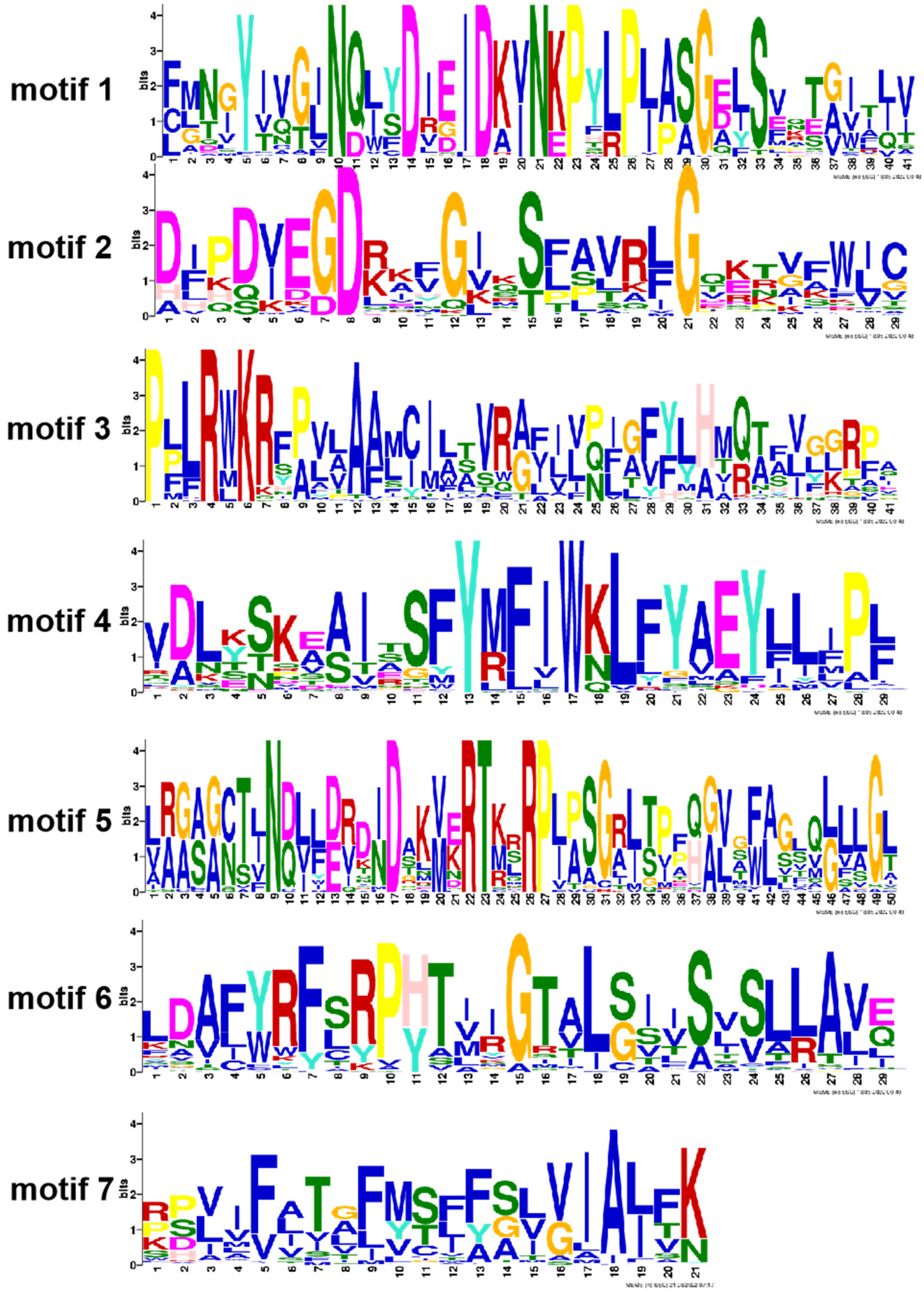

Figure S3. 7 conserved motifs of the *UBiA* genes.

The size of the amino acid indicates the frequency of occurrence in the motif, and the superimposed partial amino acids indicate the presence of other amino acid residues at that position.

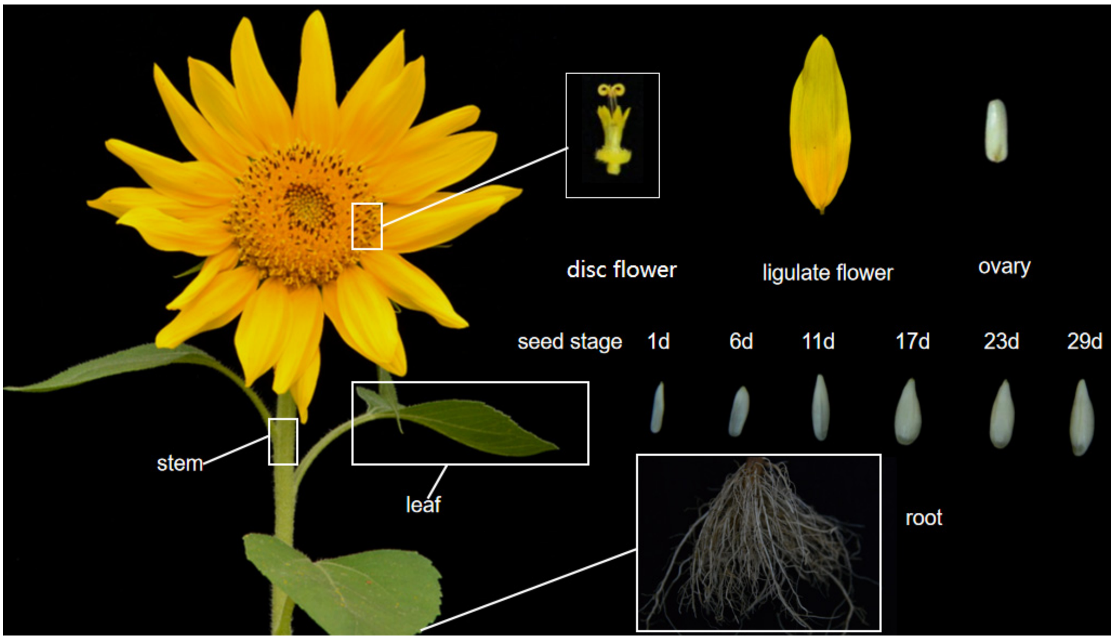

Figure S4. Photographs of differenttissues in Helianthus annuus.

Roots, stem, leaf, ovary, ligulate, disc flower, seeds at different developmental stages were showed.

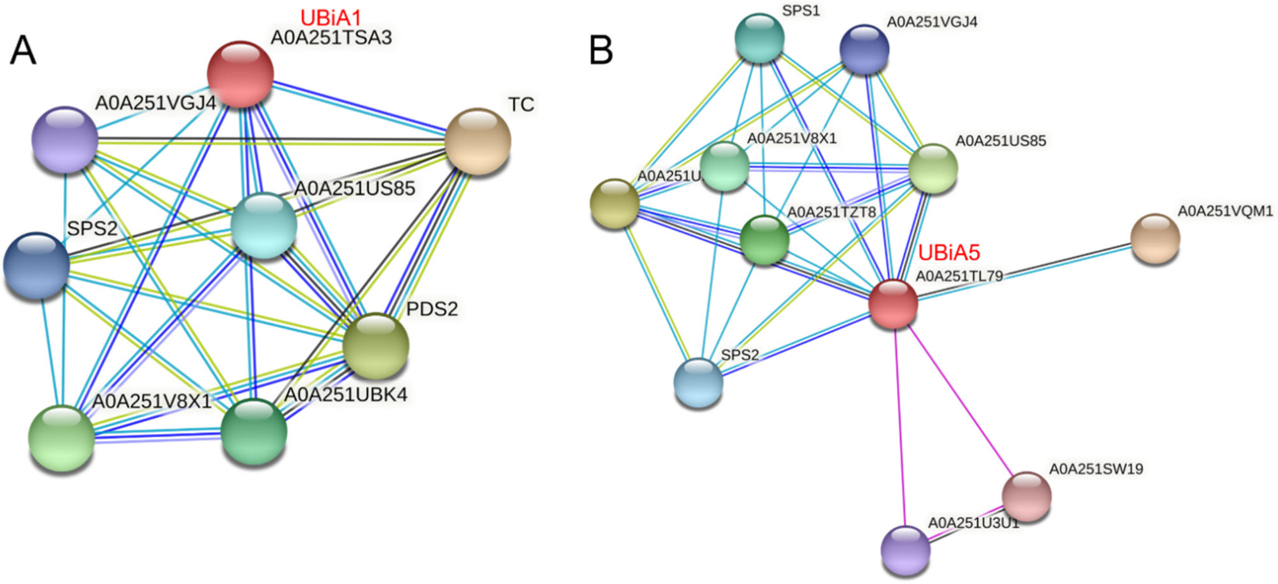

Figure S5. Functional regulatory network of HaUBiA1 and HaUBiA5.

Protein interactions of UBiA proteins were predicted using STRING software. Data from the database were indicated by green lines, experimentally determined data were indicated by purple lines, dark green indicated protein neighboring, red lines indicated protein fusion, blue lines indicate gene co-occurrence; yellow lines indicated text mining, black lines indicated co-expression, and gray lines indicated protein homology.

Table S1. Asteraceae Ka, Ks and Ka/Ks

Seq_1	Seq_2	Ka	Ks	Ka/Ks
LsUBiA2	HaUBiA6	0.08519918	0.498798352	0.170808864
LsUBiA6	HaUBiA2	0.451828163	1.733162922	0.260695724
LsUBiA12	HaUBiA5	0.12817063	0.570879613	0.224514289
LsUBiA6	HaUBiA8	0.686027038	3.180223856	0.215716588
LsUBiA11	HaUBiA4	0.11546902	0.585913641	0.197075153
LsUBiA9	HaUBiA8	0.085410128	0.567084873	0.150612602
LsUBiA13	HaUBiA2	0.218975036	0.720189146	0.304052119
AlUBiA7	HaUBiA5	0.117636898	0.468027109	0.251346333
AlUBiA4	HaUBiA4	0.116446807	0.498669385	0.23351505
AlUBiA2	HaUBiA6	0.085333091	0.368695696	0.231445856
AlUBiA6	HaUBiA8	0.078880094	0.509679724	0.154764042

Table S2. The list of sunflower *HaUBiA* genes primers used in RT-qPCR.

Name	Sequence
HaTuBulin-F	CTGATTGTCGTAAACGCTTG
HaTuBulin-R	CTCAAGATCAGCAACAGTGC
HaUBiA1-F	ATGCAGGCTCTTGTTGGAGG
HaUBiA1-R	GCAATCGAAAAGCCCATGACA
HaUBiA2-F	AATGGCGTCTCTAGCTGTGG
HaUBiA2-R	GAAGCTTGTGTTCCTGTGGC
HaUBiA3-F	TCTTCCACTTCAAACCGACG
HaUBiA3-R	CTGGTGCCTTAGCTTTAACTTCA
HaUBiA4-F	ACACGCTCAAAGGAACGGTA
HaUBiA4-R	TCGCCTGCATAACTCCAAGG
HaUBiA5-F	CTCTCTCCACTGGTGCGGAT
HaUBiA5-R	GCTTGCCCATGGGTAGTGTA
HaUBiA6-F	CTCCTCTTATGGGGTGGGCT
HaUBiA6-R	ACCCGAAGGATCAGCGAAAG
HaUBiA7-F	CACCGACTTTCTCTGGCTTTGA
HaUBiA7-R	ACGCCATTGTTGGCTAGGG
HaUBiA8-F	TTGGGTCAAAAGCGGGTGTT
HaUBiA8-R	AATCGCGTGACCCAATACCG
HaUBiA9-F	GAGTACTTGCTGGTGCCCAT
HaUBiA9-R	CCATCACATCTCACGCTTGC
HaUBiA10-F	CATGCCTCAGGCTTTCAGGA
HaUBiA10-R	CTGCAATTGCCTCCTGGGTA
